# Echoes of the embryo: using the developmental biology toolkit to study cancer

**DOI:** 10.1242/dmm.023184

**Published:** 2016-02-01

**Authors:** Nicole M. Aiello, Ben Z. Stanger

**Affiliations:** Departments of Medicine and Cell and Developmental Biology, Abramson Family Cancer Research Institute, and Institute for Regenerative Medicine, Perelman School of Medicine at the University of Pennsylvania, 421 Curie Boulevard, Philadelphia, PA 19104, USA

**Keywords:** Cancer, Embryology, Intravital imaging, Lineage tracing, Reprogramming

## Abstract

The hallmark of embryonic development is regulation – the tendency for cells to find their way into organized and ‘well behaved’ structures – whereas cancer is characterized by dysregulation and disorder. At face value, cancer biology and developmental biology would thus seem to have little to do with each other. But if one looks beneath the surface, embryos and cancers share a number of cellular and molecular features. Embryos arise from a single cell and undergo rapid growth involving cell migration and cell-cell interactions: features that are also seen in the context of cancer. Consequently, many of the experimental tools that have been used to study embryogenesis for over a century are well-suited to studying cancer. This article will review the similarities between embryogenesis and cancer progression and discuss how some of the concepts and techniques used to understand embryos are now being adapted to provide insight into tumorigenesis, from the origins of cancer cells to metastasis.

## Abstract (precis)

**Summary:** This article reviews the similarities between embryogenesis and cancer progression and discusses how the concepts and techniques of developmental biology are being applied to provide insight into all aspects of tumorigenesis.

## Introduction

The cancer research community has relied on some of the same tumor-modeling systems for decades, namely cell lines, xenografts and, more recently, genetically engineered mouse models (GEMMs). Mouse models are widely considered to more accurately represent the human tumor microenvironment compared to cell line injections and xenografts; however, even GEMMs have limitations when it comes to recapitulating the more dynamic aspects of tumor biology. For example, clonal relationships, invasive properties and cell-cell interactions are not readily apparent upon histological examination of a murine tumor, when one is staring at a field of seemingly identical cancer cells. Furthermore, GEMMs can only be used to model cancers characterized by one or a few specific genetic mutations, but many cancers, especially hematological malignancies, are caused by large-scale chromosomal rearrangements that cannot be easily replicated in a murine system. Cancer researchers have recently turned to some classic developmental biology techniques to overcome these limitations. This Review will highlight some of the common cellular and molecular mechanisms shared by developing organisms and tumors, and then provide a detailed description of how developmental biology tools, specifically lineage tracing, live imaging and cellular reprogramming, have been repurposed to study various aspects of tumor progression.

## Common themes in embryogenesis and cancer

**Fig. 1.**
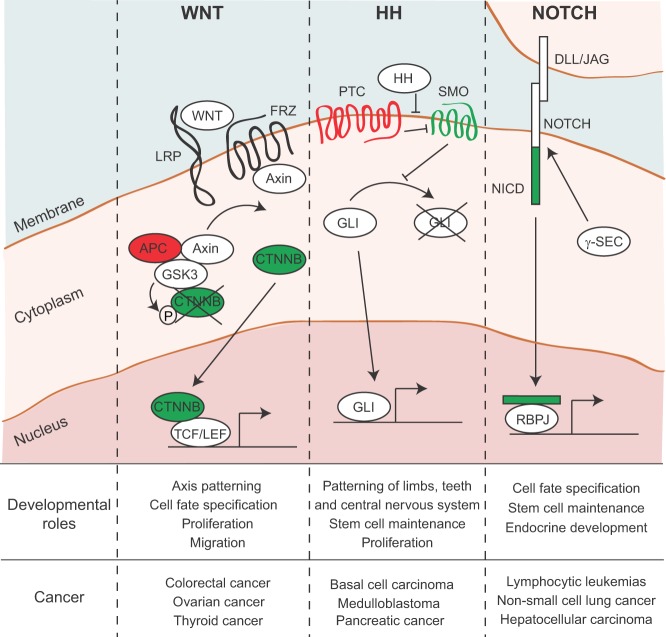
**Developmental pathways are frequently activated in cancer.** WNT, HH and NOTCH pathways are involved in many aspects of embryogenesis, including, but not limited to, patterning, cell fate specification, proliferation and stem cell maintenance. Dysregulation of each of these signal transduction pathways has been implicated in tumor initiation. In each case, the pathway is activated upon binding of a ligand to a receptor on the plasma membrane. This sets off a cascade of events allowing an effector to translocate to the nucleus and affect gene transcription. Pathway genes that are typically inactivated in cancer are highlighted in red; genes that are frequently hyperactivated in cancer are shown in green. WNT, wingless-type MMTV integration site; FRZ, Frizzled; LRP, low-density lipoprotein receptor-related protein; APC, adenomatous polyposis coli; GSK3, glycogen synthase kinase-3; CTNNB, β-catenin; P, phosphorylation; TCF, T-cell-specific transcription factor; LEF, lymphoid enhancer binding factor; HH, hedgehog; PTC, patched; SMO, smoothened; GLI, GLI-family zinc finger; DLL, delta-like; JAG, jagged; NICD, notch intracellular domain; γ-sec, γ-secretase; RBPJ, recombination signaling binding protein for kappa J region.

The process of embryogenesis requires precise spatial and temporal activation of developmental signaling pathways (Slack, 2006). Re-activation of these embryonic signals in adult cells, a consequence of mutations and epigenetic remodeling, is a characteristic feature of cancer. Key developmental signaling pathways – including the Wnt, Hedgehog and Notch pathways – are frequently dysregulated in cancer and participate in all stages of tumor progression, from initiation and maintenance to metastatic spread and growth at distant sites (Fig. 1).

During development, Wnt signaling is involved in cell fate specification, proliferation and migration, and in the adult this pathway is involved in maintaining homeostasis in tissues such as the intestine, where Wnt signaling is critical for stem cell function (Dorsky et al., 1998; Loebel et al., 2003). There are multiple Wnt pathways, including a canonical pathway that regulates gene expression, and two non-canonical pathways that regulate planar cell polarity and calcium flux (Anastas and Moon, 2013). The canonical Wnt pathway is the one that is most frequently implicated in cancer. As depicted in Fig. 1A, in the absence of a Wnt signal, the canonical Wnt effector β-catenin (CTNNB) is continuously phosphorylated and targeted for degradation by glycogen synthase kinase-3 (GSK3), which is stabilized when complexed with adenomatous polyposis coli (APC) and Axin. In response to Wnt ligands, which signal through Frizzled receptors (FRZ) and their coactivators – low-density lipoprotein receptor-related proteins 5 and 6 (LRP) – Axin binds FRZ and is prevented from stabilizing the APC-GSK3-CTNNB complex, freeing CTNNB to enter the nucleus and facilitate the transcriptional activity of the T-cell-specific transcription factor (TCF) and lymphoid enhancer-binding factor (LEF) family (TCF/LEF) (Anastas and Moon, 2013). Some of these TCF/LEF targets, in turn, drive cellular growth and proliferation, and thereby drive tumorigenesis when the pathway is hyperactivated.

Canonical Wnt signaling is active in nearly all developing tissues and plays a crucial role in body axis patterning (Petersen and Reddien, 2009), stem cell maintenance and lineage specification (see Box 1 for a glossary of selected developmental biology terms) (De Calisto et al., 2005; Sokol, 2011). In the adult, hyper-activation of the Wnt pathway – typically due to loss of the tumor suppressor protein APC – represents the first step in colorectal tumorigenesis (Giles et al., 2003). Ligand-independent activation of CTNNB is a more common alteration in cancers outside the gut, especially in endometrioid ovarian cancer, hepatoblastoma and Wilms' tumors (Bell, 2005; Polakis, 2007).

**Box 1. Glossary of developmental biology terms**

**Ectoderm:** the outer layer of the three germ layers.

**Endoderm:** the inner layer of the three germ layers; forms the lining of the gastrointestinal tract, pancreas, liver, lungs and other glands.

**Epiblast:** upper layer of the mammalian blastoderm; eventually forms the embryo proper as well as amniotic ectoderm and extraembryonic mesoderm.

**Epithelium:** a sheet of adherent, polarized cells lying on a basement membrane.

**Gastrulation:** phase during early embryogenesis characterized by dynamic cell movements when the blastula differentiates into three germ layers (endoderm, mesoderm and ectoderm).

**Mesenchyme:** motile, mesoderm-derived cells scattered throughout extracellular matrix.

**Mesoderm:** the middle layer of the three germ layers.

**Morphogen:** a secreted factor that depends on local threshold concentrations to determine cellular signaling output.

**Neural crest:** a transient, motile embryonic cell population that develops into numerous cell lineages, including melanocytes, smooth muscle, neurons and craniofacial mesenchyme.

**Parenchyma:** the functional tissue of an organ.

**Patterning:** spatial and temporal organization of cell fates.

**Pluripotent stem cell:** a cell with the ability to self-renew and produce all embryonic cell types but not extraembryonic cell types.

**Primitive streak:** thickened layer of epiblast cells that becomes mesoderm during gastrulation.

**Specification:** when a cell or tissue is committed to a particular cell fate but the decision can still be reversed.

**Stroma:** the supportive tissue of an organ, including connective tissue and blood vessels.

Adapted from *Essential Developmental Biology*, Slack (2006).

The Hedgehog (HH) pathway, like the Wnt pathway, plays a critical role in the development of many organs, including but not limited to the patterning of the central nervous system (CNS), tooth development and limb formation (Thesleff and Sharpe, 1997; Ulloa and Briscoe, 2007; Zeller et al., 2009). The three HH ligands – Sonic hedgehog (SHH), Indian hedgehog (IHH) and Desert hedgehog (DHH) – act as morphogens to direct left-right asymmetry and cell fate decisions, and pattern developing tissues (Ingham and McMahon, 2001). In the absence of HH ligands, the transmembrane protein Patched (PTC) indirectly facilitates the degradation of GLI-family zinc finger proteins (GLI) by blocking Smoothened (SMO) activity. In the presence of HH ligands, PTC is unable to inhibit SMO, which stabilizes GLI proteins and allows them to translocate into the nucleus where they transcriptionally activate genes involved in proliferation and differentiation (Fig. 1B) (Briscoe and Thérond, 2013). In the pathogenic context, activation of the HH pathway can lead to basal cell carcinoma (BCC) (a type of skin cancer) (Athar et al., 2006) or to the recruitment of a fibroblast-rich stroma in pancreatic cancer (Rhim et al., 2014; Tian et al., 2009).

The Notch pathway is similarly involved in embryonic cell fate decisions; specifically, it is crucial for the development of organs including but not limited to the CNS, pancreas, bone and heart (Andersson et al., 2011). This pathway is activated by juxtacrine (contact-dependent) signaling between the Notch receptor on the receiving cell and Notch ligands, including Delta-like (DLL) and Jagged (JAG), on the adjacent signaling cell. Upon binding of Notch ligands to the receptor, γ-secretase (γ-SEC) cleaves the intracellular Notch domain (NICD), allowing it to enter the nucleus and facilitate the transcriptional activity of Recombination signaling binding protein for kappa J region (RBPJ) (Fig. 1C) (Bray, 2006). Notch's role in tumor progression seems to be context-dependent, as the pathway can act as an oncogene in some settings (e.g. breast cancer and T-cell leukemia) or as a tumor suppressor gene in others (e.g. skin cancer) (Avila and Kissil, 2013; Bolós et al., 2007; Radtke and Raj, 2003), although the latter might be through a non-cell-autonomous mechanism (Demehri and Kopan, 2009). Other cellular signaling pathways in addition to Wnt, HH and Notch play crucial roles in cell fate specification and migration during embryogenesis, and have also been implicated in cancer invasion and metastasis. These include the fibroblast growth factor (FGF) and transforming growth factor β (TGFβ)-bone morphogenic protein (BMP) signaling pathways, which promote cell migration and invasion in both the developmental and cancer contexts (Heisenberg and Solnica-Krezel, 2008; Moustakas and Heldin, 2007; Pan, 2010; Strutz et al., 2002).

A recurring motif in development is reciprocal signaling between neighboring cell populations: crosstalk that facilitates morphogenesis of the emerging tissue. Reciprocal signaling between developing epithelium and mesenchyme (see Box 1) occurs repeatedly during embryogenesis and is crucial for the formation of limbs, epidermal appendages, pancreas, lungs, kidney and other organs (Butterfield et al., 2010; Fu and Hsu, 2013; Hrycaj et al., 2015; Landsman et al., 2011; Levinson and Mendelsohn, 2003; Li et al., 2015; Wells et al., 2013). Carcinomas also contain a mixture of epithelium (cancer cells) and mesenchyme, which, in the cancer context, is known as stroma. Cancer stroma consists of leukocytes, fibroblasts, endothelium and lymphatic vessels, which collectively form what is known as the tumor microenvironment (TME). These cell populations engage in molecular crosstalk with cancer cells, which can affect cancer cell survival, proliferation and migration. An example is provided by pancreatic cancer. As touched upon above, pancreatic tumor cells secrete SHH, a HH ligand, which recruits fibroblasts – characterized in this context as cancer-associated fibroblasts (CAFs) – to form the dense ‘desmoplastic’ or fibrotic stroma of that cancer type (Bailey et al., 2009, 2008; Tian et al., 2009). This process is known as desmoplasia. The fibroblast-rich stroma of pancreatic cancer has been demonstrated to exert both pro- and anti-tumor effects. Although desmoplasia is proposed to impair chemotherapeutic drug delivery (Jacobetz et al., 2013; Olive et al., 2009; Provenzano et al., 2012), depletion of CAFs using genetic and pharmacological methods results in increased tumor growth and metastasis (Özdemir et al., 2014; Rhim et al., 2014). Signaling between cancer cells and the stromal cells they recruit is a common theme in carcinoma progression. Through reciprocal interactions such as these, tumors build a non-cancerous stroma that in turn influences cancer cell behavior.

Embryogenesis entails dramatic morphological changes and cellular movements that are recapitulated within tumors. One of the most notable of these is epithelial-to-mesenchymal transition (EMT), a process in which epithelial cells lose their epithelial characteristics, including apical-basal polarity and cell-cell adhesion, and take on the motile features of fibroblasts. The primary role of EMT is during embryonic development, where it is crucial for gastrulation and other developmental events; however, cancer cells exploit this property of increased motility to facilitate spread. During gastrulation, WNT, TGFβ and FGF orchestrate primitive-streak formation by promoting EMT via the activation of the transcription factors Snail and Twist (Ciruna and Rossant, 2001; Heisenberg and Solnica-Krezel, 2008; Peinado et al., 2007). These developmental ‘EMT factors’ are also drivers of EMT and consequent metastasis in breast, pancreatic and colorectal cancers, among others (De Craene et al., 2005; Yang et al., 2004). EMT is again required later in development, for neural crest cell migration, and is then facilitated by Snail, Slug (also known as Snail2) and the Zeb family of transcription factors; Snail and Zeb family proteins have also been implicated in cancer cell invasion and dissemination (Cheung et al., 2005; Comijn et al., 2001; Hajra et al., 2002).

In conclusion, the molecular cues used to pattern an embryo are harnessed by tumor cells to enhance growth, recruit stromal cells and coordinate spread from the primary tumor. Because of the many commonalities between development and cancer at the molecular and cellular levels, it has been possible to use the tools of developmental biology to address fundamental questions about tumor biology. Below, we highlight some classical developmental biology techniques that have been repurposed in new and innovative ways to expand our understanding of cancer.

## The developmental toolbox repurposed in cancer

### Fate mapping

Fate mapping, also known as lineage labeling or lineage tracing, was originally developed to visualize the fate of individual cells and their progeny, referred to as clones, during embryogenesis, but it has also proven useful for the study of tumor-initiating populations and dynamic cellular movements in the context of cancer. Early fate-mapping experiments employed vital dyes, fluorescent dyes or radio-labeling to mark a specific region, lineage or even a single cell of an embryo and follow it throughout development as cells divide and migrate away from their original positions. These studies allowed embryologists to generate detailed fate maps for a number of model organisms, including frog, zebrafish and chick (Dale and Slack, 1987; Kimmel et al., 1990; Matsushita, 1996).

**Fig. 2.**
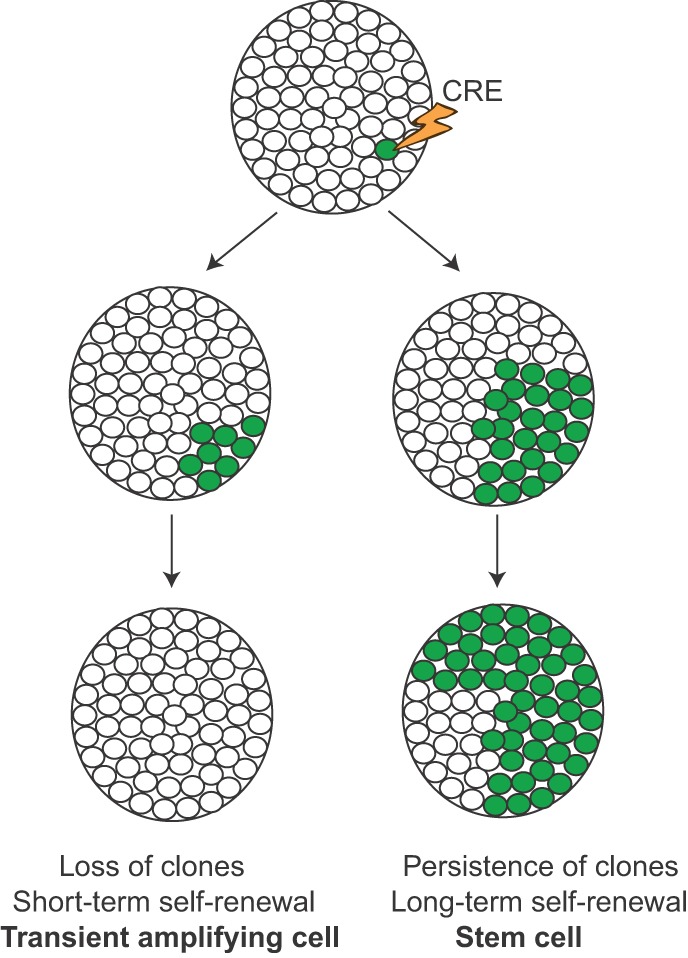
**Use of lineage labeling to identify stem cells during development and tumor progression.** Using inducible Cre-recombinase technology (Box 2), cells within a lineage are sparsely labeled to provide the resolution necessary to identify clonal populations. After a short period of time, labeled progeny (shown in green) become apparent. If the original labeled cell is a genuine stem cell, the labeled clones will persist over the lifetime of the tissue (or tumor) because the stem cell is continuously self-renewing and producing differentiated daughter cells. If, on the other hand, the labeled clones are lost over time, the original labeled cell was most likely a transient amplifying cell, which is capable of short-term self-renewal but eventually becomes terminally differentiated, no longer contributing to the pool of cells. This test is not only useful for identifying stem cells and cancer stem cells but also for detecting drug-resistant clones. After sparse labeling and chemotherapy, drug-resistant clones will persist and begin to take up a much larger fraction of the tumor cell population, much like a stem cell.

With the advent of site-specific recombinase technology, including Cre-*loxP* and flippase/flippase recognition target (FLP/*FRT*) systems, it became possible to genetically label and track a cell lineage, facilitating fate mapping in mammals, which, unlike lower model organisms, are not transparent and contain many more cells and lineages. Cre and FLP are both recombinases and, although they recognize different genetic sequences, they function similarly by inducing DNA recombination to facilitate DNA manipulation at target sites (Box 2). Of these two systems, Cre-*loxP* is more commonly used in mammalian genetics, but FLP/*FRT* is also utilized, sometimes in combination with Cre when multiple recombination events are necessary to mark a lineage within a lineage. Genetic lineage tracing, made possible by Cre technology, has been utilized to investigate the cell-of-origin for cancer, as well as to study clonal heterogeneity and metastasis, as described below and illustrated in Fig. 2.

**Box 2. Cre-based recombination**

Cre was first discovered in P1 bacteriophage, which use the enzyme to facilitate viral genome replication and to progress from the latent to lytic phase (Hamilton and Abremski, 1984). Cre recognizes specific DNA sequences (*loxP* sites) and targets these for recombination, which, depending on the orientation of the sites, can result in deletion, inversion or translocation of intervening sequences (Sternberg et al., 1981; Voziyanov et al., 1999; Nagy, 2000). If *loxP* sites are oriented in the same direction, the DNA sequence between them will be excised, if they are oriented in opposite directions, the gene between them will be inverted, and if they are located on separate chromosomes, Cre will mediate a translocation. Typically, *loxP* sites are used to delete regions of DNA, such as, for example, a gene of interest or a stop codon located upstream of a fluorescent reporter gene (Sauer and Henderson, 1988). Cre expression can be restricted to specific cell types by altering upstream promoter elements, which permits spatial control of gene expression (Araki et al., 1997). Temporal control is also important, especially if recombination in adult cells is required. Thus, Cre variants that are inactive until the necessary ligand is introduced, for example tamoxifen in the case of CreER, have been developed (Feil et al., 1996).

### Cell-of-origin

A pressing issue in cancer biology is the elucidation of tumor-initiating cells or the ‘cell-of-origin’ in cancer: which cells in a normal tissue give rise to cancer? Given the robust self-renewal capacity of cancer cells, it is often assumed that cancers arise from resident, adult stem cells within tissues, and hence the concepts of ‘cell-of-origin’ and ‘cancer stem cells’ are often conflated. (The cancer stem cell hypothesis posits that a subset of cells within the tumor harbor most of the tumor's long-term self-renewal capacity, a concept quite distinct from the cell-of-origin, which merely points to the cell type within a tissue most likely to be transformed by the initiating mutation.) Importantly, because cancer cells can, in principle, acquire stem cell properties as a consequence of mutation or epigenetic remodeling, they need not arise from stem cells. The ability of tumors to emerge in tissues in which it is questionable whether stem cells exist (e.g. the kidney) is further evidence that cancers can arise from fully differentiated cells.

Lineage tracing provides a powerful tool to identify stem cell populations in embryonic and adult tissues, and the same approach has now been used to identify tumor-initiating cells in cancer (Fig. 2). Several years ago, Lgr5 – encoded by a Wnt-target gene – was identified as a marker of intestinal stem cells: Lgr5^+^ cells labeled with Cre-based technology durably gave rise to all the differentiated cell types of the intestinal villi (Barker et al., 2007; Schepers et al., 2012). Building on this approach, Barker and colleagues used additional Cre-based tools to delete the tumor-suppressor gene *Apc* in mice in either the stem cell compartment (using Lgr5-Cre) or the non-stem-cell ‘transient amplifying’ compartment – capable of short-term self-renewal only – (using Ah-Cre, which can be induced in the gut epithelium by lipophilic xenobiotics such as β-napthoflavone) (Barker et al., 2009). Whereas *Apc* deletion in the stem cells resulted in adenomas (premalignant lesions) that exhibited unimpeded growth, *Apc* deletion in the transient amplifying cells resulted in microadenomas (tumors less than 10 mm in diameter) whose growth rapidly stalled (Barker et al., 2009). This result suggested that – in the context of an *Apc* mutation – intestinal stem cells are more competent than their transient amplifying cell progeny to form tumors.

At present, lineage tracing offers the most robust method of addressing the cancer stem cell hypothesis *in vivo*. By using tamoxifen-induced Cre systems such as CreER and titrating the dose of tamoxifen, one can limit recombination to rare, sparse cells within a tissue, providing the resolution to identify clonal populations. Utilizing this approach, Driessens and colleagues were able to identify cancer stem cells and their progeny in a chemical-induced carcinogenic model of squamous cell carcinoma (SCC) (Driessens et al., 2012). Using a lineage-labeling system driven by Keratin 14 (K14)-CreER, which marks basal epithelial cells within the epidermis, the authors found that only 20% of these cells are capable of generating a large clonal population of pre-malignant papilloma cells. However, when the authors allowed these papillomas to progress to malignant SCC, the tumors became poorly differentiated and exhibited a much higher frequency of long-term replicative cells, suggesting that, in the transition from benign to malignant tumors, the hierarchy of cancer stem cells and differentiated progeny starts to fall apart (Driessens et al., 2012).

Such lineage-tracing experiments support the general notion that cancer is the result of having the ‘right’ mutations in the ‘right’ cell at the ‘right’ time. In other words, certain cell populations (stem cells or non-stem cells) might be susceptible to the oncogenic effects of certain gene mutations that have no effect in other cell populations. Additional factors, such as the local environment (e.g. inflammation), could contribute to the susceptibility or resistance of various cell types to the cancer-causing effects of a given mutation. Similar lineage-tracing experiments have identified cells-of-origin in a number of contexts (Choi et al., 2012; Guest et al., 2014; Li et al., 2013; Liu et al., 2011; Wang et al., 2011; Alcantara Llaguno et al., 2015). From these studies, it seems that some tumors, including intestinal tumors and basal cell carcinomas, arise from resident tissue stem cells, whereas, in other cases, it seems that tumors can arise from fully differentiated cells (e.g. pancreatic tumors, cholangiocarcinomas, gliomas). Further studies are needed to elucidate the specific factors involved in determining the cell-of-origin in different cancer types.

### Clonal heterogeneity and tumor evolution

Genomic analyses have confirmed that tumors are composed of numerous ‘sub-clones’, or clones with distinct mutations in addition to the original tumor-driving mutation(s) (Greaves, 2015; Tabassum and Polyak, 2015; Grove and Vassiliou, 2014). Such tumor heterogeneity is a consequence of ‘clonal evolution’, a process whereby cells within a cancer can acquire different mutations that lead them to be genetically and phenotypically distinct. A recent study has suggested that interactions between different sub-clones might drive tumor growth (Marusyk et al., 2014), suggesting that heterogeneity might not be merely a byproduct of clonal evolution but could also underlie key features of tumor biology.

Lineage tracing lends itself to the study of clonal evolution and the complex relationships between clonal populations. One useful tool has been the ‘Confetti’ mouse, a strain in which cells are labeled with one of four fluorescent colors upon activation of Cre activity (Snippert et al., 2010). The system relies on the fact that Cre mediates different recombination events depending upon the orientation of *loxP* sites (Box 2). Within the Confetti allele, four fluorescent-protein genes are flanked by *loxP* sites and oriented in such a way that excision or inversion results in the (somewhat) random expression of one of the four lineage labels. Using this system, it has been possible to identify ‘bottlenecks’ during the clonal evolution of a tumor *in vivo*. For example, premalignant progression in pancreatic cancer from acinar-to-ductal metaplasia (ADM) to more advanced pancreatic intraepithelial neoplasia (PanIN) is accompanied by a shift from polyclonal to monoclonal lesions. This model has also revealed differences in metastatic potential between different tumor sub-clones, providing evidence for inter-clonal cooperation during tumor dissemination (Maddipati and Stanger, 2015).

Lineage tracing has also provided insight into clonal evolution following chemotherapeutic selective pressures. For example, using a Cre-inducible mouse model of glioblastoma, Chen et al. found that Nestin^+^-marked adult neural stem cells genetically labeled with green fluorescent protein (GFP) made up only a small fraction of a naïve tumor, but, upon treatment with temozolomide, an alkylating agent that is used to treat some brain cancers, these cells and their progeny expanded to become the most abundant tumor clone (Chen et al., 2012). Similarly, in a mouse model of SHH-driven medulloblastoma, rare quiescent Sox2^+^-marked adult neural stem cells were found to be resistant to anti-mitotic and SHH-targeted therapy and responsible for recurrence after treatment (Vanner et al., 2014). These studies offer an explanation for why single therapies typically fail: there is almost inevitably a resistant tumor sub-clone that will repopulate the tumor. The best strategy likely involves using multiple therapies targeting different pathways to reduce the chance of a single resistant sub-clone expanding out.

### EMT and invasion

It is comparatively easy to identify cells undergoing EMT during embryogenesis because whole cell populations (e.g. the epiblast, see Box 1) can be observed to undergo a transformation from an epithelial sheet to highly migratory fibroblast-like cells. However, it has proven to be exceptionally difficult to study cancer-associated EMT *in vivo* because, once a cancer cell undergoes EMT, it becomes indistinguishable from the surrounding non-cancerous stroma. Likewise, studying metastasis *in vivo* has been challenging, particularly in the context of a spontaneously growing tumor because each of the events that occur during metastasis – from invasion through basement membranes to growth at distant sites – involves rare stochastic phenomena that are difficult to capture experimentally.

Again, lineage tracing has proven to be a useful technique in studying these processes. For example, by introducing a yellow fluorescent protein (YFP) lineage label into a well-established GEMM of pancreatic cancer, it became possible to unambiguously identify cells that had undergone EMT because such cells still bore the lineage marker confirming their epithelial origins, despite acquiring a mesenchymal phenotype (Rhim et al., 2012).

Lineage tracing has also been used to elucidate the dynamics of metastasis. For example, Aytes and colleagues used a YFP reporter in the context of a mouse model of prostate cancer to determine the temporal occurrence of lung metastasis: at 1 month post-induction, rare single YFP^+^ cells could be observed in the lung; at 2 months, YFP^+^ micrometastases were evident; and, by 3 months, gross metastases were found in 100% of animals (Aytes et al., 2013). Similar observations have been made in pancreatic cancer, where lineage tracing has enabled the examination of the rare events involved in metastatic growth, down to single-cell resolution (N.M.A., B.Z.S. et al., unpublished data; Rhim et al., 2012). We foresee this tool being used in the future to reveal the molecular mechanisms underpinning spontaneous metastatic spread. At present, a diagnosis of metastatic cancer is essentially a death sentence; thus, there is a dire need for effective anti-metastatic treatments, especially for highly metastatic malignancies such as pancreatic cancer and melanoma. It is imperative for the cancer research community to utilize all of the tools in its arsenal to uncover currently unknown druggable metastasis-related signaling pathways and/or mutations.

## Cellular reprogramming

**Fig. 3.**
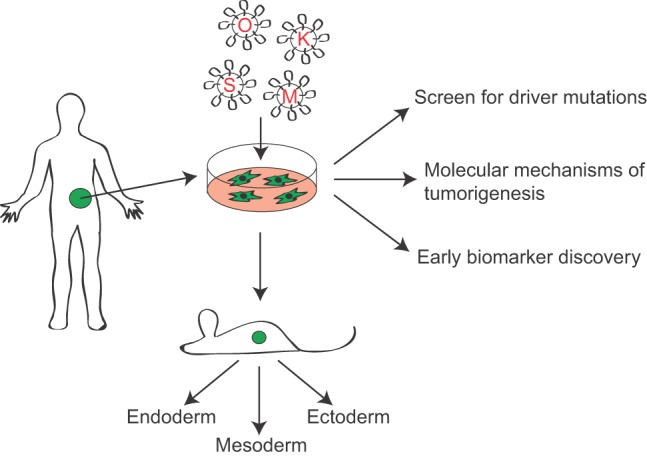
**Generation of induced pluripotent cancer cells (IPCCs).** IPCCs are derived from primary human tumor tissue by lentivirus-mediated expression of the four pluripotency factors Oct4 (O), Klf4 (K), Sox2 (S) and c-Myc (M). To confirm pluripotency, the cells are injected into immunocompromised mice and should form teratomas containing all three germ layers (Box 1). Cancer-derived iPSCs can be used to model early stages of disease or malignancies characterized by large chromosomal deletions or heterogeneous alterations that are difficult to model in transgenic mice.

The ability to generate induced pluripotent stem cells (iPSCs) has revolutionized stem cell biology and opened up new avenues for regenerative medicine. Since the initial discovery that four transcription factors can reprogram terminally differentiated cells to a pluripotent state (Takahashi and Yamanaka, 2006), investigators have refined the methods for cellular reprogramming and repurposed the technology for other applications in biomedical science. One of the more innovative uses of iPSC methodology is in modeling human cancers, particularly the premalignant stages of tumor progression that commonly go unobserved in patients (Fig. 3). Cancer-derived iPSCs offer a unique opportunity to turn back the clock and investigate how mutations direct the many phenotypic changes observed during tumor progression, including transcriptional, epigenetic and metabolic rewiring.

To date, most studies involving cancer-derived iPSCs provide proof-of-principle, demonstrating that patient-derived tumor specimens (Kumano et al., 2012) and human cancer cell lines from a myriad of cancers, including gastrointestinal malignancies (Miyoshi et al., 2010), leukemia (Carette et al., 2010), melanoma (Utikal et al., 2009), sarcoma (Zhang et al., 2013) and glioblastoma (Stricker et al., 2013), are capable of being reprogrammed to generate cell models that recapitulate the human cancer. It is only recently that cancer cell reprogramming has been taken to the next logical step of exploring the mechanisms of tumor progression, and we have highlighted most of these studies in this section.

### Molecular mechanisms of tumorigenesis

Li-Fraumeni syndrome (LFS) is an autosomal-dominant inherited syndrome driven by mutations in the *TP53* tumor suppressor gene. The disorder is characterized by early onset of a diverse range of malignancies, including osteosarcoma (OS), breast cancer and leukemia; however, the precise molecular mechanisms driving tumorigenesis in these varying tissues – bone, mammary gland and blood – are unclear. Lee et al. (2015) investigated the pathogenesis of osteosarcoma in LFS using fibroblast-derived iPSCs from individuals with LFS. They found that LFS-iPSCs failed to fully differentiate into osteoblasts *in vitro* and, unlike iPSCs derived from unaffected relatives, LFS-iPSCs formed tumors in nude mice (a commonly used model that is unable to mount an immune response). Using this tool, the authors were able to identify the imprinted *H19* locus, a gene that is crucial for osteoblast differentiation, as a target of altered p53 activity in individuals with LFS and an important repressor of OS development (Lee et al., 2015).

### Driver mutations

iPSCs also fill a void in the study of human malignancies that arises because of a lack of synteny between the mouse and human genomes. An example is provided by the myelodysplastic syndromes (MDSs): disorders of the hematopoietic system that predispose affected individuals to acute myeloid leukemia (AML) and are characterized by large chromosomal deletions. Loss of the long arm of chromosome 7 (7q) is a frequent occurrence in MDS. Genes within human 7q map to four different murine chromosomes, precluding the generation of GEMMs of MDS. To address this, Kotini et al. (2015) generated iPSCs from isogenic normal and malignant hematopoetic cells from individuals with MDS who harbor a 7q deletion. The authors were able to identify the specific disease-causing haploinsufficient genes within this locus through a series of rescue experiments. Four genes were identified along 7q that could restore hematopoietic differentiation, including *EZH2*, which is frequently mutated in MDS. Cellular reprogramming thus allowed the authors to accurately model the human disease from its early stages and zero in on the functionally important genes within the 7q locus.

### Early biomarker discovery

Although many cancers, such as colon and breast cancer, have well-defined and readily detectable pre-malignant stages, the precursor lesions associated with other cancers, such as pancreatic adenocarcinoma (PDA), are hard to detect, resulting in a lack of early screening methods. As noted above, PDA is thought to arise via ADM and the generation of PanINs, histological lesions that are not readily seen by imaging. Hence, it has been nearly impossible to identify biomarkers associated with early pancreatic disease in humans, and thereby to diagnose PDA early enough to slow its aggressive progression.

Recently, Kim et al. sought to address this unmet need by generating iPSCs from primary PDA surgical specimens and matched normal pancreatic tissue (Kim et al., 2013). Although the tumor cells were less efficient at generating iPSCs compared to normal cells, the authors were nevertheless able to create iPSC-like cell lines from a single tumor by maintaining low-level expression of the pluripotency factors. When injected into immunodeficient mice to form teratomas (benign tumors composed of all three germ layers), the tumor-derived iPSCs had a higher propensity to form endoderm (Box 1) compared to control pluripotent cells. Importantly, these teratomas formed pre-malignant PanIN lesions after 3 months, which progressed to frank PDA between 6 to 9 months, recapitulating the early steps of human PDA formation. The authors then identified a panel of secreted factors released by tumors at the 3-month time-point, providing potential biomarkers for early diagnosis. If these biomarkers prove useful for early detection it will represent a huge advance for PDA, which is typically diagnosed after it is already too late.

### Modeling pediatric cancers

So far, this section of the Review has primarily focused on the generation of pluripotent cells from cancer cells; however, the reverse process, generating cancer cells from pluripotent cells, has recently been exploited to produce a much-needed model for studying diffuse intrinsic pontine glioma (DIPG), an aggressive pediatric brain-stem cancer (Funato et al., 2014). Surgery is rarely an option for individuals with DIPG because of its anatomical location and propensity for local invasion, so clinical samples are difficult to obtain and thus only a handful of DIPG cell lines currently exist (Schroeder et al., 2014). The mean age of onset for DIPG is 8 years, suggesting that there is a relatively small developmental window during which the progenitor cell population is transformed. To generate a model for this challenging cancer, Funato et al*.* (2014) took advantage of a well-defined neural differentiation protocol for human embryonic stem cells (hESCs). After generating neural progenitor cells (NPCs), the authors transformed them with lentiviruses containing the most frequently implicated genetic alterations in DIPG: constitutively active platelet-derived growth factor receptor α (PDGFRα), histone H3.3 with a lysine-to-methionine alteration at residue 27 (K27M) and a p53 short-hairpin RNA (shRNA) knockdown construct. They found that the triple combination of lentiviruses (termed P5K) significantly increased the proliferation of NPCs, but not undifferentiated hESCs or differentiated astrocytes. The P5K condition also prevented NPCs from differentiating into glial cells, maintaining them in a progenitor state with high self-renewal capacity. When injected orthotopically into immunocompromised mice, P5K-NPCs were capable of forming tumors that had a similar histopathology to low-grade DIPG. Importantly, the authors were able to use these hESC-derived DIPG-like cell lines to perform a small-molecule screen and found that these cells were particularly sensitive to inhibitors of a tumor suppressor named Menin both *in vitro* as well as *in vivo* (Funato et al., 2014). The current standard-of-care for DIPG is radiotherapy, with a 2-year survival of only 10% (Schroeder et al., 2014), so the finding that these tumors seem to have a targetable weakness could change the game for children diagnosed with DIPG.

Collectively, the iPSC-based studies discussed above demonstrate the many ways in which our understanding of pluripotency and directed differentiation can be used to study cancer in a different light. Although the use of iPSC technology in cancer research is in its infancy, we foresee its utility in uncovering molecular mechanisms of tumorigenesis in other difficult to model cancers and pre-malignant stages.

## Live imaging

Although dynamic cellular movements are common during development, embryologists have historically relied on fixed embryos, which only offer snapshots that must be pieced together to give a cohesive view of the movements that occur *in vivo*. Recently, live imaging has become an important tool for studying development because it reduces the need for inference and allows researchers to directly follow cellular behavior in real time. Live imaging has elucidated the dynamics of neural crest cell migration in chick embryos (Birgbauer et al., 1995), vascular development in zebrafish (Lawson and Weinstein, 2002) and neural tube closure in mice (Massarwa and Niswander, 2013), and the technique is now being applied to address fundamental problems in cancer biology through intravital microscopy (IVM).

### Cell-cell interactions, movement and response to chemotherapy

Standard epifluorescence microscopy is sufficient to visualize cellular events in small transparent embryos, whereas imaging cells within living tumors requires deeper tissue penetration. In recent years, a number of modifications to traditional IVM – in particular multiphoton microscopy (MM) – have permitted real-time observations of tumors, including cell-cell interactions within primary tumors, migration of cells to metastatic sites, and chemotherapeutic responses. Two-photon excitation microscopy, the most commonly used type of MM, uses two long-wavelength, low-energy photons to excite a fluorophore, resulting in less autofluorescence, significantly reduced phototoxicity and deeper tissue penetration (up to 1 mm) compared to confocal microscopy (Ustione and Piston, 2011) (see Box 3).

**Box 3. Multi-photon microscopy and intravital imaging**

Multi-photon microscopy, and in particular two-photon microscopy, is typically used for intravital imaging. Unlike traditional fluorescence microscopy in which one photon causes a fluorophore to emit a single fluorescent photon, in multi-photon microscopy two photons simultaneously interact with a fluorophore, resulting in non-linear excitation and brighter emission. Multi-photon microscopy thus requires a particular light source, i.e. a pulsed laser, and an objective with a high numerical aperture to increase the chances of two photons hitting the same target (Ustione and Piston, 2011). In addition to detecting fluorescent reporters, multi-photon microscopy can be used to visualize specific endogenous molecules using second harmonic generation (SHG). Second harmonic light is generated by molecules with a noncentrosymmetric structure, such as collagen fibers, and is half the wavelength and twice the frequency of the light source (Campagnola, 2011). SHG is useful for generating contrast and locating landmarks during intravital imaging.

Another obstacle to long-term intravital tumor imaging is anatomic location. In particular, imaging tumors within the abdominal cavity or beneath the skull is difficult not only because of relative inaccessibility but because of artifacts introduced by animal respiration. These issues have been addressed by constructing durable imaging windows in anesthetized animals (Kienast et al., 2010; Ricard et al., 2014; Ritsma et al., 2012) and by coordinating image capture with the timing of respiration (Looney et al., 2011).

These advances have provided a more dynamic picture of the tumor microenvironment than had been surmised from fixed tissue sections. For example, IVM has revealed that leukocytes traffic in and out of primary tumors and interact with each other and cancer cells, facilitating invasion and metastasis (Wyckoff et al., 2007). In recent work by Zomer et al. (2015), the authors demonstrated extracellular vesicle exchange between tumor cells in an orthotopic mouse model of breast cancer, leading to enhanced metastatic behavior. In this study, highly metastatic MDA-MB-231 breast cancer cells were transfected with Cre and injected orthotopically along with weakly metastatic cells that were transfected with a Cre-inducible DsRed-to-GFP switchable reporter. Using IVM, the authors were able to visualize the release of extracellular vesicles by the MDA-MB-231 cells. In turn, these vesicles (which contained Cre) were taken up by the weakly metastatic cells, resulting in a switch from DsRed to GFP expression (Zomer et al., 2015).

IVM has also been used to investigate the cellular response to chemotherapy in living tumors. For example, Nakasone et al. (2012) observed doxorubicin-induced apoptosis in mammary tumors in a breast cancer mouse model (MMTV-PyMT) in real time by using propidium iodide (PI) as a reporter of cell death and found that early-stage carcinomas were the most sensitive. Importantly, cancer cells from different tumor stages did not differ in sensitivity *in vitro*, ruling out cell-intrinsic mechanisms for resistance. Returning to the intravital model, the authors found that, upon doxorubicin administration, myeloid cells were recruited to sites of necrosis, where they facilitated remodeling of nearby tumor blood vessels, causing them to become less leaky and therefore less efficient at drug delivery, sparing nearby tumor cells (Nakasone et al., 2012).

### Metastatic colonization

Live imaging has also been used to investigate metastatic colonization, a notoriously difficult stage of tumor progression to study. The metastatic cascade begins in the primary tumor, where cancer cells first invade into the stroma and make their way into the circulation. Once the cell reaches a distant organ, it extravasates into the parenchyma (Box 1), where it must survive and proliferate in a foreign, potentially hostile, microenvironment (Gupta and Massagué, 2006).

Many questions remain about the cellular and molecular mechanisms governing the establishment of micrometastatic lesions, and intravital imaging has provided some insight. For example, Kienast et al. (2010) found that the first step in colonization in a model of brain metastasis was arrest at blood vessel branches, after which a small fraction of tumor cells (1-2%) extravasated into the surrounding tissue. Only cells that achieved successful extravasation and maintained close physical contact with the endothelium went on to form micrometastases (Kienast et al., 2010). In contrast, invading cancer cells were more mobile in a mouse model of liver metastasis. Aided by an abdominal imaging window (a titanium ring with a glass coverslip), which allowed for long-term (>24 h) tracking of disseminated cells, Ritsma et al. observed a ‘pre-micrometastatic’ stage characterized by considerable migration away from liver sinusoids. Using a pharmacological inhibitor of cofilin (a regulator of the actin cytoskeleton) to prevent migration, the authors demonstrated that blocking this highly motile pre-micrometastatic stage significantly reduced the number of metastases but did not affect previously established lesions (Ritsma et al., 2012). These results suggest that therapies targeting migratory cancer cells could prove useful for preventing metastatic colonization but will most likely fall short of effectively treating established metastases. It is therefore crucial to elucidate the molecular mechanisms involved in the maintenance of metastatic lesions to advance the development of suitable therapies. Further examples of how live imaging and IVM have been used to study metastasis are reviewed in Fein and Egeblad (2013).

## Conclusions and future outlook

In this Review, we have highlighted ways in which the tools of developmental biology are providing insight into tumor biology, enabling efforts to understand the cellular origins of cancer, delineate mechanisms of tumor maintenance and metastasis, and identify early cancer biomarkers. These insights have enabled researchers to address some of the most pertinent questions in cancer research, and could pave the way for the development of new diagnostic and prognostic markers, and potential anti-cancer therapies. However, all of these techniques have limitations and room for improvement. For example, lineage-traced GEMMs, which typically harbor cancer-causing mutations throughout an entire lineage from early developmental time-points, do not fully recapitulate human carcinomas because these are thought to arise from single transformed adult cells. This is beginning to be addressed using inducible Cre systems, which can be titrated to induce recombination of the relevant transgenes in only a small fraction of cells. The trade-off is that tumor incidence will almost surely be reduced and tumor latency increased under these circumstances. An advance in this realm is a mouse genetic system called Mosaic Analysis with Double Markers (MADM) (Zong et al., 2005). The most recent iteration of MADM uses Cre-driven interchromosomal recombination, multiple *loxP* variants and two fluorescent proteins, green (GFP) and red (RFP), to sparsely label cells in a mosaic fashion, resulting in four potential recombination outcomes: GFP^+^ mutant, RFP^+^ wild type, double-positive heterozygote and colorless heterozygote (see Henner et al., 2013 for more details). This model system has not yet been widely adopted by the cancer community but it has already yielded insight into the cell-of-origin in glioma (Liu et al., 2011).

iPSC technology has only just begun to be applied to questions of cancer biology and tumor progression. It is becoming apparent from numerous cancer-iPSC studies that primary tumor cells are significantly less efficient at generating iPSCs compared to untransformed cells (Ramos-Mejia et al., 2012), a problem that could potentially be addressed by the developmental biology and stem cell communities. iPSCs are known to exhibit ‘epigenetic memory’ of their cellular origin and this can reduce the efficiency of reprogramming (Kim et al., 2010; Papp and Plath, 2013). Cancer cells harbor extensive aberrant epigenetic changes, including histone modifications and widespread DNA methylation (Dawson and Kouzarides, 2012), so perhaps the addition of an epigenetic modifier could improve the reprogramming efficiency of cancer cells (Apostolou and Hochedlinger, 2013; Ramos-Mejia et al., 2012). The epigenetics of reprogramming is not only interesting from a technical perspective but also from a biological one. Keeping in mind that cellular reprogramming essentially resets the epigenome, one potentially fascinating area of focus is the sequence of epigenetic events following oncogene activation. How do genetic mutations fundamentally restructure the epigenetic landscape to promote tumorigenesis in human cells?

Live imaging and IVM are now being used to illuminate the cellular dynamics of tumor progression and metastasis. However, there is an unmet need for autochthonous GEMMs that allow for real-time metastasis monitoring. One recently developed imaging tool is a new reporter mouse that permits noninvasive whole-animal imaging by employing the far-red Katushka fluorescent protein (KFP) (Diéguez-Hurtado et al., 2011). This strain has been used as a lineage label to track events at the cellular level in developmental and stem cell studies (Castellana et al., 2014; Isern et al., 2014; López-Iglesias et al., 2015; Trakala et al., 2015), and it is easy to imagine how introducing an inducible *KFP* allele into a cancer GEMM might permit investigators to track metastatic cells in real time. This type of technology would vastly improve our ability to vet anti-metastatic therapies by helping to identify metastasis-bearing animals and monitor their progress during treatment.

As tools such as lineage labeling, cellular reprogramming and live imaging are improved and further harnessed to provide new insight into a variety of human diseases, cancer – with its close relationship to developmental biology – will likely remain at the top of the list.
